# Argon-based geochronology: advances, limitations and perspectives

**DOI:** 10.1093/nsr/nwaf277

**Published:** 2025-07-08

**Authors:** Su-Chin Chang, Wenbei Shi, Yinzhi Wang, Fei Wang

**Affiliations:** Department of Earth Sciences, The University of Hong Kong, Hong Kong 999077, China; Institutional Center for Shared Technologies and Facilities, Institute of Geology and Geophysics, Chinese Academy of Sciences, Beijing 100029, China; State Key Laboratory of Lithospheric and Environmental Coevolution, Institute of Geology and Geophysics, Chinese Academy of Sciences, Beijing 100029, China; Innovation Academy for Earth Science, Chinese Academy of Sciences, Beijing 100029, China; Institutional Center for Shared Technologies and Facilities, Institute of Geology and Geophysics, Chinese Academy of Sciences, Beijing 100029, China; State Key Laboratory of Lithospheric and Environmental Coevolution, Institute of Geology and Geophysics, Chinese Academy of Sciences, Beijing 100029, China; Innovation Academy for Earth Science, Chinese Academy of Sciences, Beijing 100029, China; State Key Laboratory of Lithospheric and Environmental Coevolution, Institute of Geology and Geophysics, Chinese Academy of Sciences, Beijing 100029, China; Innovation Academy for Earth Science, Chinese Academy of Sciences, Beijing 100029, China; College of Earth and Planetary Sciences, University of Chinese Academy of Sciences, Beijing 100049, China

**Keywords:** K–Ar dating, ^40^Ar/^39^Ar dating, advances, limitations, perspectives

## Abstract

Given that K constitutes about 3 wt.% of Earth's crust and is present in most rock-forming minerals, and that Ar diffusion in minerals is temperature-dependent, Ar-based geochronology (^40^Ar/^39^Ar and K–Ar dating) can date most rocks and also reveal their thermal history. This paper reviews recent advances and longstanding limitations in ^40^Ar/^39^Ar and K–Ar geochronology, and provides perspectives into future research on Ar-based geochronometers. Over the past two decades, multi-collector noble gas mass spectrometry has witnessed remarkable advancements in both sensitivity and resolution. Successive upgrades of mass spectrometer generations have significantly enhanced the precision of Ar isotope measurements, enabling a comprehensive revision and optimization of ^40^Ar/^39^Ar dating standard minerals. To achieve high-precision ^40^Ar/^39^Ar dating and minimize inter-laboratory discrepancies, researchers are focusing on refining the potassium decay constant, developing standardized mineral separation techniques, and harmonizing irradiation and data processing protocols. These efforts are pivotal for improving the analytical precision of low-K and young samples, thereby expanding the application frontiers of ^40^Ar/^39^Ar geochronology. For *in*  *situ* planetary dating, the K/Ar method currently remains the only feasible radiometric technique among radioactive isotope systems. Addressing challenges in simultaneous K and Ar measurements will facilitate streamlined acquisition of reliable datasets. Moreover, research is advancing toward a deeper understanding of Ar diffusion behavior in minerals—beyond temperature-dependent volume diffusion—to clarify its impact on ^40^Ar/^39^Ar data interpretation and geological significance. To further advance argon-based geochronology, the scientific community is committed to continuous exploration and resolution of methodological limitations inherent in these dating approaches.

## INTRODUCTION

In recent decades, the K–Ar and ^40^Ar/^39^Ar dating techniques have emerged as highly effective geochronological tools that are utilized across a range of disciplines within the Earth sciences, including tectonics, biostratigraphy, volcanology, planetary geology, ore mineralization and hydrocarbon generation [1–13].

The ^40^Ar/^39^Ar dating technique is an advance on the K–Ar dating method, whereby the K content of a sample is determined based on the ^39^Ar produced via the nuclear reaction ^39^K (n,p) ^39^Ar during irradiation. This method facilitates the simultaneous measurement of both the parent (^40^K) and daughter (^39^Ar) isotopes using a single noble gas mass spectrometer. Theoretically, the ^40^Ar/^39^Ar technique is capable of mitigating sample heterogeneity, thereby providing age estimates that are more precise than K–Ar dating. However, some uncertainties, such as interference from other nuclides, recoil effects and neutron fluence gradients, complicate the acquisition of high-precision ^40^Ar/^39^Ar ages [14–17]. Consequently, a comprehensive understanding of the reaction processes that occur during irradiation is necessary to minimize these interferences. Standards are used to monitor neutron irradiation processes. The utilization of reliable and homogeneous mineral standards at the scale of a single grain is essential for accurate age determinations.

In contrast to the ^40^Ar/^39^Ar dating method, the precision of age determinations using the K–Ar dating method is relatively low due to the separate analysis of K and Ar on different aliquots of a sample. However, the K–Ar method has some advantages over ^40^Ar/^39^Ar dating. For example, it does not require sample irradiation and the analyses are relatively quick. Although the conventional K–Ar method has some inherent disadvantages, advances have been made in the unspiked K–Ar dating method and simultaneous K and Ar analysis techniques [18–21]. Consequently, K–Ar dating offers distinct advantages for *in situ* dating of planetary surfaces, as well as for fine-grained samples. The advent of a new generation of multi-collector noble gas mass spectrometers has significantly advanced the precision and accuracy of Ar isotopic analysis [22].

Advances in dating techniques are dependent on ongoing improvements in instrumentation and methodologies. This paper reviews recent developments in instrumentation, methodologies, standards and applications of the K–Ar and ^40^Ar/^39^Ar dating methods over the past two decades. More importantly, the longstanding limitations of Ar-based dating methods are discussed. This paper will be of interest to the ^40^Ar/^39^Ar dating community and researchers who use other geochronometers, and also geoscientists interested in understanding K–Ar and ^40^Ar/^39^Ar dating.

## NOBLE GAS MASS SPECTROMETRY

Application of the ^40^Ar/^39^Ar method has progressed in tandem with advances in noble gas mass spectrometry. This has resulted in enhanced sensitivity, improved precision and higher sample throughput, as well as enabling the analysis of smaller amounts of material. These advances have occurred alongside international collaborations, such as the EARTHTIME initiative, which have promoted high-precision ^40^Ar/^39^Ar and other types of dating, and its application to various geological processes.

The ^40^Ar/^39^Ar dating method requires the analysis of five Ar isotopes (^36^Ar, ^37^Ar, ^38^Ar, ^39^Ar and ^40^Ar). As the gas is introduced into the mass spectrometer, the intensities of the Ar signals decrease over time. In traditional single-collector mass spectrometers, sequential measurement of each isotope allows for the construction of regression lines of the isotope ion beam intensity versus time. This process facilitates the determination of the isotopic composition at the moment of gas introduction, which is commonly referred to as the ‘time-zero’ composition. Due to the varying abundances of the Ar isotopes, each isotope exhibits a unique decay trend that is affected by its partial pressure in the mass spectrometer. This variability represents a potential source of error. To mitigate this, multi-collector mass spectrometers, which were developed about two decades ago, enable the simultaneous collection of multiple ion beams, and thus can measure isotopic compositions to a higher precision [23].

The introduction of gas from extraction or purification systems into a mass spectrometer requires tens of seconds to reach equilibrium. During this period, the gas within the mass spectrometer begins to be consumed. The definition of time-zero varies between laboratories, specifically regarding the interval from gas introduction to the start of measurements. This variability has the potential to introduce discrepancies in measurements in different laboratories. Recently, a novel emission suppression technology has been developed that allows for the suspension of sample consumption until the samples have equilibrated within the mass spectrometer. This advance might reduce the variations in results that arise from inconsistencies in the definition of time-zero, and improve the accuracy and precision of noble gas isotope ratio measurements.

Faraday collectors and electron multipliers are the primary signal collectors in noble gas mass spectrometers. While the Faraday collector is well known for its stability, it is limited in its ability to effectively measure small ion signals, such as those of <1 mV. Conversely, the electron multiplier can detect small ion signals. However, it is subject to limitations due to the dead time and quasi-simultaneous arrival effects, which restrict its optimal working range. Exceeding this range can lead to deviations from signal linearity, which can affect the data accuracy. In ^40^Ar/^39^Ar dating, the abundances of ^40^Ar and ^39^Ar are much greater than those of the other three Ar isotopes. It is standard practice to measure these isotopes using different types of collectors, which leads to the need to correct for the efficiency of the different collectors. The recent introduction of 10^13^ Ω amplifiers allows Faraday collectors to detect signals as low as 0.1 mV, with an internal precision of better than ±0.5%. Although this technological advance does not completely replace the electron multiplier, it does enable the simultaneous measurement of the five Ar isotopes with Faraday collectors and enhances the measurement stability. In turn, this increases the accuracy and precision of age determinations. This technology has been incorporated into several types of noble gas mass spectrometers [24–26].

Noble gas mass spectrometers can be classified according to their focus on either high resolution or high sensitivity. Attaining a high resolution requires the use of larger magnets and extended flight tubes, which in turn increase the overall volume of the spectrometer. This can adversely affect the sensitivity of the instrument. Consequently, it is challenging to attain both high resolution and high sensitivity simultaneously with a single noble gas mass spectrometer. Given this limitation, it is prudent to select different mass spectrometers that are specifically engineered to meet particular measurement requirements. In summary, advances in customized mass spectrometers capable of attaining a high resolution without sacrificing sensitivity, as well as the design of mass spectrometers that mitigate the effect of human factors, represent potential avenues for future improvements in noble gas mass spectrometry.

## METHODOLOGY

### K–Ar dating

The limitations of separately measuring K and Ar contents on different sample aliquots, with different techniques, have led to the K–Ar method being replaced gradually by the ^40^Ar/^39^Ar method. However, in some cases, K–Ar dating is still a useful technique. Over the past 30 years, the unspiked K–Ar technique has been developed, which has advantages over the conventional K–Ar dating technique.

Conventional K–Ar dating involves spiking with ^38^Ar, and can be used for samples that are difficult to date by the ^40^Ar/^39^Ar method due to issues arising from neutron irradiation when analyzing fine-grained or Fe-rich samples. Loss of ^39^Ar due to recoil during neutron irradiation makes the ^40^Ar/^39^Ar method unsuitable for dating fine-grained minerals. Similarly, the significant production of highly radioactive nuclides (^51^Cr, ^59^Ni, ^58^Co and ^54^Mn) from Fe during neutron irradiation of Fe-rich samples is a radiation hazard to the analyst and makes such samples unsuitable for ^40^Ar/^39^Ar dating. However, unspiked K–Ar dating enables the ^38^Ar/^36^Ar ratio of the sample to be accurately determined. By comparing this ratio with that of the atmospheric standard, the initial ^40^Ar/^36^Ar ratio in the sample can be determined. As such, the unspiked method is ideal for dating young (≤1 Ma) volcanic rocks.

Unspiked K–Ar dating has the potential to be used with the *in situ* laser probe technique, which would allow K–Ar isochrons to be constructed. Coupled with laser-induced breakdown spectroscopy (LIBS), unspiked K–Ar dating enables the simultaneous measurement of K and Ar on a single laser spot on a sample [21]. Given that K is a major and widely distributed element, this might facilitate remotely controlled field dating, including materials on the surface of a planetary or asteroidal body on a space mission.

The advantages of *in situ* laser probe unspiked K–Ar dating are as follows: (i) the simultaneous measurement of K and Ar, which avoids the issue of sample heterogeneity; (ii) the potential to determine spatial variations in age; and (iii) its applicability to minerals that are fine-grained or difficult to separate from the sample. However, simultaneous analysis of the solid element K and noble gas Ar presents significant challenges. Accurate determination of the Ar content requires a high-vacuum environment (∼10^–10^ Torr), necessitating K measurements under the same conditions. However, at a vacuum of <10^–4^ Torr, the atomic emission intensities become much lower, which compromises the analytical precision for K [19]. Therefore, obtaining high-precision K measurements under high-vacuum conditions is still challenging and requires varying the laser energy and ablation based on differences in the K concentration and age of the sample. Jesús Solé determined ages using the *in situ* K–Ar dating method applied to micas of known age (940–70 Ma). The deviations of the ages from the conventional K–Ar ages were <5% for most samples [27]. Cattani *et al*. developed and calibrated a complete *in situ* K–Ar dating procedure (KArMars) based on LIBS and quadrupole mass spectrometry, which confirmed the reliability of the measurements for Martian analyses based on analysis of terrestrial analogs [20]. Wang *et al*. proposed a new method for planetary surface dating during deep space exploration, which is the *in situ* unspiked K–Ar method [28]. This paper describes in detail the recent advances and existing limitations of this technique, and makes some suggestions as to how it might be improved.

Another possible *in situ* unspiked K–Ar dating method is the simultaneous measurement of K and Ar in the plasma liberated from the sample by a laser, and using the same optical spectroscopy technique. Compared with the well-established technique for K measurement by spectrometry, the analysis of small amounts of Ar by spectrometry is challenging and needs to be refined.

In general, the K–Ar dating method has a shorter analysis period and avoids irradiation-induced interferences due to the absence of neutron irradiation as compared with ^40^Ar/^39^Ar dating. Therefore, the former is the preferred choice for dating high-Ca, Fe-rich and fine-grained samples. However, the K–Ar dating method has limited accuracy and precision due to the separate measurement of K and Ar. The *in situ* laser probe unspiked K–Ar dating method can overcome these drawbacks, and is applicable to remotely controlled field dating (e.g. on a deep space mission).

### ^40^Ar/^39^Ar dating

The ^40^Ar/^39^Ar method has some longstanding problems, most of which are due to neutron irradiation of the samples and standard monitors during the analyses. Recoil loss of ^37^Ar and ^39^Ar can be greater than expected, but the use of appropriate standards can prevent these uncertainties from propagating into the resultant ages. These problems, and progress in their resolution, are reviewed in this section.

In the ^40^Ar/^39^Ar method, the sample to be dated is first irradiated in a nuclear reactor to transform ^39^K to ^39^Ar through interactions with fast neutrons. Compared with the K–Ar method, the ^40^Ar/^39^Ar method eliminates the problem of sample heterogeneity and can potentially yield ages that are more accurate. In addition, the incremental heating technique can identify excess ^40^Ar and ^40^Ar loss, using inverse isochron and age spectra diagrams [29].

**Figure 1.**
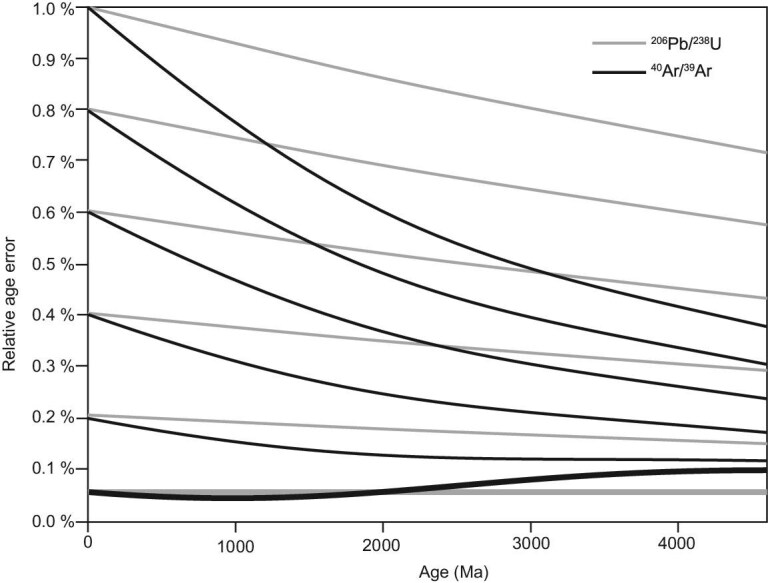
Summary comparing the accuracy of ^40^Ar/^39^Ar ages with the ^238^U/^206 ^Pb system [31]. Relative age errors estimated are shown for values of R with relative precision between 0% and 1.0%. The relative uncertainty of ^40^Ar/^39^Ar ages significantly improves with increasing age using new estimates of λ_ε_ and λ_β_ values. Relative age errors refer to the proportional uncertainties associated with age determinations, expressed as a percentage of the measured age. They quantify the precision of dating results by indicating how much the true age is likely to deviate from the measured value relative to the age itself.

To improve the precision of Ar-based chronological data, there have been advances in instrument development (Noble gas mass spectrometry section), identifying age standards (New standard monitors section) and refining the K decay constant [22,30–32]. The decay constants for the branched decay of ^40^K due to electron capture (λ_ε_) and β^–^ emission (λ_β_) have significant systematic errors. Renne *et al*. used a statistical optimization approach to obtain new λ_ε_ and λ_β_ values that improved the uncertainties on these decay constants [31]. As shown in Fig. 1, this resulted in a significant improvement in the relative uncertainties on ^40^Ar/^39^Ar ages with increasing age. Using existing techniques, the ^40^Ar/^39^Ar dating system can yield ages with accuracies comparable to or even better than those of the ^206^Pb/^238^U system. These improvements in instruments, standards, decay constants and analytical procedures have enabled ^40^Ar/^39^Ar dating to determine ages with an analytical precision of ±0.05%. However, the neutron irradiation has a significant effect on the age precision, which is discussed in the following section.

#### Interfering nuclides

During neutron irradiation, additional isotopes of Ar are generated from Ca, K, Ar and Cl [29,33]. By carefully selecting the irradiation time and shielding, these interferences can be minimized, keeping the associated errors below 1% [34]. However, in extreme cases, such as young samples with low K/Ca ratios, interference reactions, especially those involving Ca, may limit the age precision. The interference corrections can by determined by measuring the Ar isotopes produced by pure Ca and K salts irradiated under the same conditions as unknown samples and neutron flux monitoring.

The interference reaction leading to the production of ^36^Ar is critical, because ^36^Ar determines the atmospheric Ar content of samples. Carter *et al*. identified a previously overlooked production pathway: ^39^K (n,α,β) ^36^Ar. This interference becomes more pronounced with increasing time between irradiation and analysis (Δ_tirr_), and increasing age difference between the unknown sample and neutron flux monitor [33]. This highlights the importance of minimizing Δ_tirr_ and matching the ages of unknown samples and neutron flux monitors during ^40^Ar/^39^Ar dating [33].

Furthermore, ^80^Kr (^80^Kr^2^^+^) is an isobar of ^40^Ar [35]. Phases with low K and high Br content (e.g. some amphiboles), as well as samples with abundant fluid or melt inclusions, are prone to ^80^Kr^2^^+^ interference. This interference can lead to inaccuracies in ^40^Ar/^39^Ar dating results, ranging from the per mil to % levels. In step-heating experiments, ^80^Kr^2^^+^ behaves similarly to excess ^40^Ar. This interference can be monitored by the parallel reaction ^81^Br (n,β^–^) ^82^Kr.

#### Recoil of ^37^Ar and ^39^Ar

Recoil loss of ^37^Ar and ^39^Ar during irradiation can also affect ^40^Ar/^39^Ar dating results, particularly for fine-grained materials such as meteorites, glauconite and cryptocrystalline rocks [36–39]. Several studies have reported that the recoil effect is approximately proportional to the grain length and thickness [14–16]. For some important applications of ^40^Ar/^39^Ar dating that analyze crystals with sizes less than the apparent grain size threshold, an appropriate d_0_ value (available for sanidine and biotite) and the approximate crystal dimensions can be used to effectively correct for ^39^Ar loss. ^37^Ar recoil can also significantly affect the calculated age of Ca-rich minerals (e.g. plagioclase and hornblende). In contrast to ^39^Ar recoil, ^37^Ar recoil leads to an erroneously low ^40^Ar^*^/^39^Ar_K_ ratio and, consequently, produces an apparent age that is younger than the true age. In practice, the recoil effects from irradiation can be evaluated and corrected for based on the mineral composition and size.

#### Neutron fluence gradients

The neutron fluence distribution is important in determining the uncertainty on the J value (one of the major uncertainties in ^40^Ar/^39^Ar dating). Neutron fluences in nuclear reactors are heterogeneous [17,40]. Rutte *et al*. reported that radial neutron fluence gradients can be up to 2%/cm, even with rotation during irradiation [17]. Radial flux monitoring is essential for high-precision ^40^Ar/^39^Ar dating, even in fast-rotating irradiation facilities. Metallic Ni foils are useful for assessing spatial variations in the fast neutron flux during sample irradiation. In addition to conventional fission spectrum neutrons, Rutte *et al*. designed and tested a compact deuteron–deuteron (D–D) fusion neutron generator with nearly monoenergetic neutrons, which can reduce collateral nuclear reactions and improve the prediction of recoil energies [10]. D–D neutron generators could expand the applicability of ^40^Ar/^39^Ar dating methods to fine-grained materials and lead to future improvements in irradiation techniques.

### New standard monitors

For ^40^Ar/^39^Ar dating, standards are used to assess the experimental procedure and calculate the age of the unknown samples. As such, efforts have been made to develop high-quality standards. Some initially developed standards are now considered unsuitable for use because of their heterogeneous ages at the single-grain level and limited availability. However, some standards, such as Fish Canyon sanidine (FCs) and Alder Creek sanidine (ACs), yield uniform and reproducible single-grain ages, and are now commonly used standards in ^40^Ar/^39^Ar chronology [25,30,41]. In addition, the astronomically calibrated FC age of 28.201 Ma has an unprecedented accuracy and precision [28,42,43]. To date unknown samples of variable age and chemical composition with higher accuracy, new standards need to be identified and characterized. The following is a brief review of the minerals proposed in the past decade as potential ^40^Ar/^39^Ar dating standards.

Precambrian reference materials are essential for the dating of Precambrian geological and extraterrestrial samples. The 2.7 Ga NL-25 hornblende, 2.6 Ga WA1ms muscovite, 2.0 Ga BSP-1 hornblende, 1.8 Ga ZMT04 muscovite and 1.1 Ga Hb3gr hornblende have been used as standards. Compared with the hornblende standards, muscovite has a high K_2_O (10 wt.%) and low Ca content, which has the following advantages: (i) it allows the analysis of very small amounts of muscovite while still yielding sufficient ^40^Ar (and ^39^Ar) for analysis; (ii) it requires no post-irradiation waiting time; and (iii) it minimizes the need for Ca-related interference corrections. As such, the muscovite standard is suitable for most uses.

Currently, the most commonly used Precambrian standard is Hb3gr hornblende (1.08 Ga), which exhibits good inter-grain age reproducibility and has an old age [44]. However, the Hb3gr hornblende is rich in Ca and Cl, which increases the effects of interfering isotopes. Jourdan *et al*. presented a new 2.61 Ga secondary muscovite standard, which is WA1ms [45]. The flat age spectra and relative standard deviations of the average F value of 0.43%–0.60% (*P* = 0.15–0.83) show that WA1ms is a suitable ^40^Ar/^39^Ar standard for Precambrian samples. We used an R_[WA1ms/FCs]_ value to calculate a preferred age of 2613.0 ± 2.4 Ma (±0.09%; 1σ) for the WA1ms standard. In addition to the development of new reference standards, the ZMT04 muscovite standard has been recalibrated. Zhang *et al*. reported a new age of 1772.2 ± 2.7 Ma (2σ; MSWD = 1.30; *P* = 0.18) for the ZMT04 muscovite standard. Compared with previous results, the precision of this age determination has been significantly enhanced. Consequently, the ZMT04 muscovite is a reliable reference material for ^40^Ar/^39^Ar and K–Ar dating of Paleoproterozoic geological samples.

High-quality Mesozoic and Paleozoic standards are relatively scarce. Some previously proposed mica and amphibole standards were found to be potentially heterogeneous at the single-grain level. As a result, new standards have been proposed in recent years. Machlus *et al*. reported an assessment of sanidine from the Fire Clay tonstein as a Carboniferous ^40^Ar/^39^Ar monitoring standard [46]. Single-grain ^40^Ar/^39^Ar analyses (*n* = 263) of this sanidine have internal 2σ uncertainties at the ±1 Ma level (±0.3%), with ages ranging from 315 to 317 Ma (∼1% precision) and an average age of 315.36 ± 1.10 Ma (2σ total uncertainty). The excellent single-crystal reproducibility suggests this sanidine has the potential to be a reference material for Paleozoic samples. For the Mesozoic standards, Zhang *et al*. reported on sanidine from the Yunshan caldera complex (Fujian Province) and its potential as an Ar geochronological standard [47]. The Yunshan sanidine has homogeneous K (7.95 ± 0.18 wt.%) and Ca (0.15 ± 0.01 wt.%) contents, and yielded an age of 93.74 ± 0.31 Ma (2 standard deviation [SD]) at the single-grain level [47]. Wang *et al*. presented a new Cretaceous standard (i.e. Qingshan sanidine [QSs]) from the Jiaolai Basin, China. *In situ* major element data revealed it has high and uniform K content [48]. The QSs yielded a weighted-mean age of 121.71 ± 0.29 Ma (0.24%; 2σ).

In contrast, there are sufficient well-characterized Cenozoic standards. In addition to the FCs and ACs, Wang *et al*. presented a sanidine standard from a phonolite from Yabachi, Tibet, China (YBCs). The YBCs also has homogeneous K content, ^40^Ar^⁎^/^39^Ar_K_ ratios (F values) and ages at the single-grain level. The calibrated age of the YBCs is 29.286 ± 0.206 Ma [49].

The development of new reference materials can facilitate intercalibration between various reference materials and provide more options for different unknown samples. However, there are still many issues regarding the use of reference materials that need to be resolved (Calibration and development of standards section).

## APPLICATIONS OF Ar-BASED CHRONOLOGICAL METHODS

As one of the most widely used isotopic chronometers, the ^40^Ar/^39^Ar dating method is employed in almost all geological fields. In this section, some widespread applications are discussed, including those related to biological evolution, mineralization, sedimentation, planetary science and the dating of very young samples.

### Biological evolution

Since the early 21st century, ^40^Ar/^39^Ar geochronology has enabled the precise dating of several key evolutionary events. This method has provided crucial information regarding the age of the most primitive avian dinosaurs from the Jurassic Yanliao Biota [8,50], the development of the tropical rainforest biome associated with the mid-Miocene Zhangpu amber biota [51], the earliest hominid fossils discovered in Pliocene strata in East Africa [52] and the global migration patterns of later *Homo* species. While direct dating of fossils using the ^40^Ar/^39^Ar method is not possible, accurate age determinations of interbedded volcanic ash or lava flows associated with fossil-rich beds have enhanced our understanding of past ecosystems and biodiversity. The combination of geochronological and paleontological research has enabled the evaluation of hypotheses relating to various aspects of the evolution of life on Earth.

The ^40^Ar/^39^Ar method has had a key role in accurately dating significant sites connected to human evolution, including bone beds, human footprints and stone tools. These age determinations have enhanced our understanding of the chronological framework of hominid evolution. Renne *et al*. [53] obtained ages of 5.6–3.9 Ma for the Sagantole Formation in the Afar Rift by ^40^Ar/^39^Ar dating of 12 volcanic units, with a resolution of better than ±100 ka. This framework confirmed an age of 4.4 Ma for *Ardipithecus ramidus*, or ‘Ardi’, which is the oldest hominid skeleton yet discovered, which predates *Australopithecus afarensis* (Lucy) by 1.2 Ma [53,54]. This precise dating offers insights into the evolutionary timeline from our common ancestor with chimpanzees through to early human genera and species [55,56].

Fossilized human footprints are rare, but provide key insights into the evolution of bipedalism. ^40^Ar/^39^Ar dating has played a key role in this field of research. Renne *et al*. [34] dated basaltic tuff containing footprints to 1.30 ± 0.03 Ma, challenging existing ideas on the timing of migration into the Americas. Scaillet *et al*. dated the ‘Devil's footsteps’ in Italy to 0.345 ± 0.006 Ma. The oldest footprints in Europe, attributed to *Homo heidelbergensis*, date to the middle Pleistocene and represent a species that followed *Homo erectus* [57].

^40^Ar/^39^Ar dating also provides insights into stone tool development by early humans, which is crucial for studying human evolution. The shift from Acheulean stone tool-making to the Middle Stone Age was a pivotal moment characterized by significant technological advances and cultural traits linked to the emergence of *Homo sapiens* [58]. Deino *et al*. used ^40^Ar/^39^Ar dating to establish the chronology of Acheulean and early Middle Stone Age deposits in the Olorgesailie Basin, in the southern Kenya Rift Valley [59]. They found that late Acheulean tool assemblages yielded ages of 615–499 ka. A significant transition occurred at *ca*. 320 ka, marking the emergence of the Middle Stone Age, which lacked Acheulean elements. Hominins at these sites made cores and points, and sourced stone materials from 25–50 km away, highlighting changes in their interactions with the environment and each other, which in turn provided insights into human social and cognitive development [60].

### Challenges in dating sedimentation

The K–Ar and ^40^Ar/^39^Ar dating methods have been utilized for stratigraphic age determinations for many years [61,62]. Authigenic clay minerals, especially illite and glauconite, along with K-feldspar overgrowths, can be used to date diagenesis in sedimentary rocks. However, recent findings indicate that the ages obtained from these minerals are mainly younger than those obtained from other methods such as lithostratigraphy, paleomagnetism and biostratigraphy, leading to skepticism about the reliability of ^40^Ar/^39^Ar and K–Ar ages. It is increasingly clear that diagenetic processes, mineral formation and fluid metasomatism affect the K–Ar isotopic system. Consequently, ^40^Ar/^39^Ar and K–Ar dating of sedimentary rocks requires careful sample preparation and characterization.

Illite is a key target for the dating of sedimentary rocks, because its grain size shows a close correlation with its age. Framework grains can cause illite to have erroneously old ages. As such, a repetitive freezing and thawing method has been proposed to mitigate the effect of framework grains on illite age determinations [61]. Furthermore, various crystallinity indices, including the crystallinity index, Kuber index and illite polytype identified by X-ray powder diffraction, can differentiate the illite source or generation, thereby enhancing the interpretation of the age data. Clauer *et al*. distinguished two illite generations that crystallized under different thermal and chemical conditions using various mineralogical analysis methods, δ^18^O data and K–Ar dating, rather than attributing the older illite generation to having a detrital source [61]. These findings indicate that successive thermal and diagenetic events affected Cambrian‒Ordovician oil-bearing strata in the Hassi Messaoud area, which show that hydrocarbon trapping occurred during the Late Cretaceous and the second stage of illite authigenesis.

Bermúdez-Chávez *et al*. reported an innovative illite K–Ar dating technique [63]. They used a combination of LIBS and mass spectrometry to undertake *in situ* laser K–Ar dating, and the results were comparable to traditional bulk K–Ar ages. This method requires only milligrams of illite and can simultaneously measure the K and Ar content. Furthermore, the deposition of illite particles onto circular disks facilitates X-ray diffraction analysis, thereby optimizing the procedure. The K and Ar content of well-characterized illite samples enable such materials to be used as internal standards. The application of matrix-matched standards in conjunction with unknown samples is critical to ensuring the accuracy and reliability of the dating results.

A major challenge in using the ^40^Ar/^39^Ar method for illite dating is recoil [16,64]. Although the loss of ^39^Ar due to recoil can be effectively addressed by vacuum encapsulation [39], this approach offers minor improvements in accuracy and increases the sample preparation costs, limiting the widespread application of this method. A new generation of D–D fusion neutron generators may reduce nuclear recoil effects and allow successful dating [7], but their fast neutron flux of ∼3 × 10^7^ n/cm^2^/s [65] is much lower than the flux of 0.08–14 × 10^13^ n/cm²/s of conventional swimming pool reactors [29]. Consequently, D–D sources require longer irradiation times, making it challenging to date older samples.

The extended duration of irradiation complicates the correction for interfering reactions, especially for Ca, which generates substantial quantities of ^36^Ar and ^39^Ar. The generation of ^37^Ar (*t*_½_ = 34.95 ± 0.04 days) via the reaction ^40^Ca (n,α) ^37^Ar is minimal under atmospheric conditions, making it a key tracer for assessing the Ca content in samples and corrections for ^36^Ar and ^39^Ar [29]. When a sample is irradiated in a swimming pool reactor with a fast neutron flux of 0.08 × 10^13^ n/cm²/s for a duration of 10 min, it fulfills the analytical requirements for the production of ^39^Ar. Conversely, a D–D reactor requires about 185 days, during which 73.6% of the ^37^Ar decays to ^37^Cl, potentially leading to errors in the ^36^Ar correction and underestimation of the ^40^Ar/^39^Ar age, particularly in samples with low K/Ca ratios. Furthermore, most unknown samples need a fast neutron fluence of >10^13^ n/cm^2^ [29]. As such, the K–Ar dating method remains the preferred choice, yielding reliable illite age estimates when proper sample separation and pretreatment are applied [61].

### Dating of mineralization

Accurately dating the timing of mineralization and hydrocarbon formation is a significant challenge because of the complexity of the processes involved and subsequent alteration.

For solid minerals, suitable materials for K–Ar (^40^Ar/^39^Ar) dating can be readily found in host rocks, veins and minerals associated with ore mineralization, such as alunite [66], micas (including biotite, muscovite, sericite and phlogopite [67]), albite [68], K-feldspar [6,69], amphiboles [70] and lamprophyre and diabase veins [71]. To tightly constrain the timing of mineralization, numerous studies have conducted ^40^Ar/^39^Ar dating of fluid inclusions in quartz [72,73]. Qiu *et al.* have advanced high-precision fluid inclusion ^40^Ar/^39^Ar geochronology, developing a unique method combining stepwise crushing of inclusions with step-heating of the residual solid minerals [74]. This has been successfully applied to the investigation of ore deposits. The published experimental results demonstrate that the stepwise crushing method effectively separates primary from secondary inclusions. The ages of the primary inclusions are within the error of the ages of the step-heated solid residue. There has been significant progress in ^40^Ar/^39^Ar dating of fluid inclusions in quartz and ore minerals such as wolframite, cassiterite and sphalerite [1,9,10,74–76].

Direct dating of ore minerals using the ^40^Ar/^39^Ar method remains exploratory in nature. Sphalerite and pyrite, which are common in metallic ore deposits, are promising target minerals [74,77]. K-bearing Mn oxides have been used to date supergene deposits [4,78,79]. The coronadite-group minerals and romanechite are also suitable for ^40^Ar/^39^Ar geochronology, as they can host structural K [80]. Not all Mn oxides are suitable for ^40^Ar/^39^Ar dating. The K content in Mn oxides varies significantly and is affected by both the composition of the weathering solution and the crystal structure of the minerals. Therefore, the key factor for obtaining reliable ^40^Ar/^39^Ar ages of Mn oxides is the ability of the minerals to effectively retain K and its radiogenic decay product ^40^Ar [79].

The ^40^Ar/^39^Ar dating method has also been used to determine the timing of gas and oil accumulation. Mark *et al*. showed that the growth rims of diagenetically formed K-feldspar are important chronological markers for both diagenetic processes and associated hydrocarbon migration events [12]. The high spatial resolution of ultraviolet laser microprobe ^40^Ar/^39^Ar dating, when applied to authigenic K-feldspar, effectively mitigates the effects from detrital minerals. This methodological advance is a powerful tool for constraining the maximum age of oil and gas emplacement [11,12]. Furthermore, quartz grains in volcanic rocks hosting gas reservoirs can provide chronological constraints, as they are ideal for precise dating if they contain abundant secondary fluid inclusions that have high trapped concentrations of K and elevated methane partial pressures due to gas emplacement [13].

With ongoing research and development, it might be possible to determine ore mineralization ages using Ar-based geochronology by combining indirect methods (e.g. dating alteration minerals and veins) with direct methods (e.g. dating ore minerals). However, the extremely low K content, presence of excess ^40^Ar, and multi-stage inclusions in ore minerals are problems that need to be solved in future studies.

### Planetary sciences

The ^40^Ar/^39^Ar (including K–Ar) dating technique has been used to investigate the early evolution of the solar system [81,82]. A decade ago, this method was successfully utilized for *in situ* dating on Mars, making it the first successful *in situ* dating of an extraterrestrial planet [18]. Despite challenges in mineral separation and gas extraction, the age of the Sheepbed mudstone of 4.21 ± 0.35 Ga indicates that K–Ar dating is currently the only available technique for extraterrestrial *in situ* dating.

Researchers are continually improving extraterrestrial *in situ* dating methodologies and exploring techniques for obtaining ages for meteorites that are more accurate. Several factors can strongly affect the initial Ar content of meteorites, including the atmospheric Ar composition in the meteorite source region, production of ^38^Ar and ^36^Ar through cosmic ray spallation during transit, and post-landing contamination by atmospheric Ar [83,84]. Previous investigations of Martian meteorites have indicated that numerous samples have ^40^Ar/^39^Ar ages that exceed those determined by U–Pb, Rb–Sr, Lu–Hf or Sm–Nd dating methods [3]. Cassata and Borg found that conventional methods for subtracting cosmogenic nuclides are inadequate for Martian samples because of their inherent complexities [84]. They developed an enhanced correction method for cosmogenic nuclides in Martian meteorites, building on prior methodologies and insights from K/Ca ratio determinations during ^40^Ar/^39^Ar step-heating experiments. Using this method, they reanalyzed the NWA4468 and NWA2975 meteorites, yielding isochron ages of 188 ± 17 and 184 ± 17 Ma, respectively, consistent with ages from other isotopic systems. Cohen *et al*. applied this refined methodology to seven additional shergottites from Mars with anomalous ^40^Ar/^39^Ar ages, and obtained ages consistent with other isotopic chronometers, thereby validating this method [3]. Despite challenges in accurately estimating contributions from elements other than K and Ca (e.g. Fe, Ti, Ni, Mn and Cr) in calculating cosmic ray exposure ages, this refinement undoubtedly enhances our ability to date Martian meteorites and other samples affected by atmospheric and cosmogenic ^36^Ar.

Meteorites are often analyzed using the whole-rock step-heating ^40^Ar/^39^Ar dating technique, given the difficulty in isolating sufficient quantities of a pure mineral separate. This approach frequently yields disturbed age spectra that complicate a straightforward interpretation. Boehnke *et al*. found that the Ar diffusion parameters of clinopyroxene and feldspar intersect at high temperatures in Arrhenius plots [85,86]. This indicates a transition from a less-retentive phase at lower temperatures to a more-retentive phase at higher temperatures, and vice versa. Such variations in Ar diffusivity in mineralogical systems contribute to the disturbed age spectra obtained from whole-rock analyses. This allows the whole-rock dating results to be modeled with a multi-activation energy and multi-diffusion domain approach [86], and allows for the dating of minerals within meteorites and reconstruction of their thermal history [82].

Multi-activation energy and multi-diffusion domain modeling of the Jilin chondrite (K-4) and Apollo 16 lunar breccia (sample 67514,43) suggests that disturbed age spectra can form from an instantaneous impact event, with the duration of microseconds to tens of seconds being dependent on temperature [86]. These findings highlight that the apparent ages of disturbed age spectra in meteorites result primarily from the differential loss of Ar from various minerals under transient high-temperature and high-pressure conditions. Although these ages may lack clear geological significance, evidence of subsequent impact events in meteorites can be identified. Jourdan *et al*. successfully isolated pure plagioclase, pyroxene and groundmass from meteorites originating from the asteroid 4 Vesta for ^40^Ar/^39^Ar dating [82]. Their analysis revealed that the pyroxene, derived from equilibrated basaltic eucrites, is younger than the plagioclase, suggesting that the samples experienced a late impact event that disturbed the Ar isotopes of pyroxene while leaving plagioclase unaffected. This indicates that Vesta had magmatic activity for 33 ± 5 Ma following its formation. By integrating the ^40^Ar/^39^Ar ages of four equilibrated eucrites with the peak metamorphic temperature and ages of the samples, Jourdan *et al*. calculated a cooling rate of 17.3 ± 3.6°C/Ma [82]. These findings emphasize the significance of Ar diffusion in minerals and the ^40^Ar/^39^Ar dating method for understanding extraterrestrial magmatism, metamorphism, cooling processes and impact events.

### Dating very young volcanoes

The ages of volcanic rocks can be used to determine their tectonic setting, volcanic eruption histories, potential future volcanic activity and volcanic hazards. While ^40^Ar/^39^Ar dating is an important tool for dating volcanic eruptions, there are challenges in applying this approach to young volcanoes. The primary challenge in ^40^Ar/^39^Ar dating of young volcanic rocks stems from the exceedingly low abundance of radiogenic ⁴⁰Ar in samples. Consequently, the precise measurement of ^36^Ar for subtracting atmospheric ^40^Ar contamination becomes indispensable. Using a cold trap to enrich the gas can increase its concentration and improve the accuracy of the data. Mass discrimination and background corrections must rely on rigorously characterized and reproducible parameters. For young specimens, short irradiation durations combined with cadmium shielding to mitigate thermal neutron flux have effectively minimized the influence of (^40^Ar/^39^Ar)_K_ corrections on age calculations. Finally, implementing a quaternary-aged neutron fluence monitor narrows the dynamic range of isotopic ratios requiring measurement, thereby alleviating potential complications from peak tailing or detector nonlinearity.

Recently, high-resolution ^40^Ar/^39^Ar dating has enhanced our capacity to date recent volcanic activity. The dating of volcanic events was advanced by the work of Renne *et al*. [34], who obtained a precise age for samples from the 79 AD eruption of Mount Vesuvius. This ‘historic’ calibration was undertaken on sanidine. Other notable examples of the successful implementation of this approach include dating of the Hualalai Volcano on Hawaii [87,88], Changbaishan Volcano in northeast China [89], two monogenetic volcanoes in the Newer Volcanic Province of southeastern Australia [90] and Ruapehu Volcano at the southern end of the Taupo Volcanic Zone in New Zealand [91]. ^40^Ar/^39^Ar dating of the Hualalai and Changbaishan volcanoes enhanced our understanding of mantle plumes and offered insights into their recent eruption history.

Yang *et al*. undertook ^40^Ar/^39^Ar dating of recent eruption material from Tianchi at the Changbaishan Volcano in northeast China [89]. The multi-aliquot step-lasering method yielded reliable ages, even for sanidine. Notably, variations in apparent ages were indicative of xenocrystic contamination [89]. Although Yang *et al*. could not obtain a plateau age for the latest pumice eruptions, they determined inverse isochron ages of 1 and 4 ka [89]. The results suggest greater volcanic activity in the past 20 ka than previously thought, raising the possibility that the risk of volcanic hazards has been underestimated [92].

## FUTURE PERSPECTIVES

### Decay constant

The total ^40^K decay constant directly affects the calculated ^40^Ar/^39^Ar and K–Ar ages. Over several decades, numerous studies have proposed various decay constants, based on different methods, such as ^40^K activity data compilations and numerical optimization (Table 1) [93–100]. The decay constant first reported by Steiger and Jäger, which has been the most widely used for decades, was derived from a compilation of ^40^K activity data. However, this value might not adequately account for the natural variability of ^40^K/K ratios in silicates and other K-bearing minerals [100]. Subsequently, the decay constants recommended by Min *et al*. [94] and Renne *et al*. [32] have been widely adopted. However, both have certain limitations. Min *et al*. [94] omitted the electron capture decay to ground state and this decay constant is less precise than that of Steiger and Jäger. The FCs ages obtained using the decay constant of Renne *et al*. [32] differ significantly from astronomically tuned ages and are generally older. Most recently, Carter *et al*. [100] used a Bayesian calibration to obtain a total decay constant of 5.5042 ± 0.0054 × 10^–10^ year^–1^, which leads to consistency amongst ^40^Ar/^39^Ar, U–Pb and astronomical chronometers. However, the results of the Bayesian calibration still exhibit minor discrepancies. For example, the nominal mean value of the ^235^U decay constant in the model is slightly higher than that in previous studies.

**Table 1.** Summary of the published total decay constant (λ_tot_) with values and uncertainties.

Value ± 1 σ (×10 ^−^^10^ yr ^−^^1^)	Reference
5.543 ± 0.010	[93]
5.463 ± 0.054	[94]
5.554 ± 0.013	[95]
5.554 ± 0.013	[96]
5.531 ± 0.014	[32]
5.529 ± 0.019	[97]
5.474 ± 0.019	[98,99]
5.5042 ± 0.0054	[100]

Calculated ages for the same sample using different decay constants yield different results. Moreover, the effects of different decay constants on ages may vary for samples of different geological ages. Table 2 provides the ages of the same sample obtained using different decay constants [100]. The results indicate that, for younger samples, the ages obtained using the values of Carter *et al*. [100] are the youngest, those obtained using the values of Renne *et al*. [32] are the oldest, and those obtained using the values of Min *et al*. [94] are intermediate in age. For older samples, the ages derived from the values of Min *et al*. are the oldest [94]. Therefore, when calculating ages, we should specify the decay constant used. In the future, the adoption of a consistent decay constant by all laboratories will enable straightforward age comparisons. Additional efforts are needed to constrain ^40^K decay constants with greater precision.

**Table 2.** Some standard ages calculated by different decay constants [100].

Sample	Age ± 1σ (Ma) Min *et al*. [94]	Age ± 1σ (Ma) Renne *et al*. [32]	Age ± 1σ (Ma) Carter *et al*. [100]
Bishop Tuff sanidine	0.7639 ± 0.0008	0.7665 ± 0.0011	0.7635 ± 0.0007
Alder Creek sanidine	1.1850 ± 0.0010	1.1890 ± 0.0016	1.1843 ± 0.0008
Fish Canyon sanidine	28.201 ± 0.0383	28.294 ± 0.0474	28.183 ± 0.035
GA-1550 biotite	99.440 ± 0.1168	99.738 ± 0.1500	99.368 ± 0.102
Hb3gr hornblende	1081.5 ± 2.791	1080.1 ± 1.4884	1079.2 ± 1.078
NL-25 hornblende	2662.5 ± 16.09	2651.8 ± 10.45	2652.2 ± 10.13

### Calibration and development of standards

The ^40^Ar/^39^Ar dating method relies on the ages of standards (as neutron fluence monitors), making it directly dependent on the accuracy and precision of these ages. The age of a standard can be determined through two approaches: (i) directly by conventional K–Ar dating of primary standards (e.g. GA1550 biotite, MMhb-1 hornblende and NL-25 hornblende); or (ii) indirectly by ^40^Ar/^39^Ar intercalibration with a primary standard for secondary standards (e.g. FCs). Secondary standards can subsequently be used to calibrate higher-order standards (e.g. ACs calibrated against FCs). As such, errors on reference material ages propagate hierarchically.

Currently, apart from a few standards calibrated with astronomically tuned ages, most have significant uncertainties on their ages. Moreover, with the reduction in sample sizes and improvement in instrumental performance, the ages of many standards have begun to show some heterogeneity. Therefore, the overall uncertainty of the age of an unknown sample is partially constrained by the uncertainty of the age of the fluence monitor.

For hornblende standards that are suitable for calibrating low-K and high-Ca samples, irradiation produces fewer target nuclides and more interfering nuclides. This leads to larger age errors on the standards, which in turn introduces greater uncertainties on the ages of unknown samples calibrated against these hornblende standards. Despite efforts to develop new reference materials, there are few dating standards that are widely used, with the mineral types and their ages being limited. Ideally, a standard should be selected to approximate the age and/or composition of the unknown sample, thereby minimizing the range of measured Ar isotopic ratios. Therefore, expanding the range of standards of different types and ages is a key area for future work. In addition, further calibration of the ages of existing standards is required. It is essential to focus on the primary sources of the age uncertainties, including heterogeneity in the irradiation fluence and mineral composition, uncertainties on the decay constants, and interference corrections.

### Isobaric interferences

The Ar isotopes can be affected by various interferences, primarily from hydrocarbons, hydrogen chloride and C_3_ compounds [101]. Accurate measurement of these interferences is essential for accurate ^40^Ar/^39^Ar age calculations, particularly for high-precision dating or when analyzing young samples. Amongst these interferences, the separation of H^35^Cl from ^36^Ar requires the highest resolution, with m/Δm = 3938 [102,103]. These interferences can be effectively resolved through the implementation of pseudo-high-resolution on a standard noble gas mass spectrometer or by enhancing the physical mass resolution of the spectrometer.

Achieving a high physical mass resolution necessitates the use of larger magnets and extended flight tubes in the mass spectrometer. Currently, spectrometers designed for noble gas analysis are focused on enhancing their mass resolving power to meet this objective. Although research on isobaric interferences in Ar isotopic measurements has been conducted and their effect on standard dating is understood, further investigations are needed [104].

### *In situ* laser ablation technique

Heating, crushing and *in situ* laser ablation are three widely utilized techniques for gas extraction from geological samples in the context of ^40^Ar/^39^Ar dating. The use of an ultraviolet laser for *in situ*  ^40^Ar/^39^Ar dating enables high spatial resolution whilst minimizing the thermal effects on the peripheral areas of the ablation pits. This approach offers a unique perspective on the interpretation of ^40^Ar/^39^Ar ages and their geological significance [105]. After mineral formation, numerous events can modify the mineral composition or structure, thereby affecting the isotopic systems. For example, Ar transport through minerals is controlled by volume diffusion, which leads to the concept of the closure temperature and thermochronological research. However, in specific contexts, samples that have undergone deformation or metamorphism yield ages that may be inconsistent with this concept. Inclusions in minerals and the dissolution–precipitation processes involved in mineral formation can make the isotopic system open, as the dissolution rate is several orders of magnitude higher than the rate of volume diffusion, even at 300°C, which is a temperature commonly associated with most geological deformation/metamorphic events [106].

Samples collected from deformed rocks commonly yield disturbed age spectra when subjected to the step-heating method, primarily as a result of the presence of mixed mineral phases, even within individual grains. By using petrological mapping, we can obtain detailed information on intra-grain chemical or microstructural variations. This information can then be utilized to select appropriate areas for conducting the laser ablation analyses.

Due to the typically low Ar content in geological samples, it is essential that the laser spot size provides a sufficient ion signal for reliable results. However, it is still possible to link isotopic data with mineral phases. These methods can better identify the relationship between geological processes and the ages determined in the laboratory.

In summary, while Ar-based geochronology is a powerful and widely used tool, it does have some limitations that necessitate future international and interdisciplinary collaborations to enhance the technique.

## CONCLUDING REMARKS

To facilitate the comparison of data from different laboratories, a uniform experimental procedure for use in the Ar-based geochronological community should be adopted, including sample pretreatment, irradiation, gas extraction, instrumental analysis and data processing. Greater international collaboration is needed in the near future.
